# The commitment to ending leprosy

**DOI:** 10.2471/BLT.25.293767

**Published:** 2025-05-01

**Authors:** Takahiro Nanri

**Affiliations:** a Sasakawa Health Foundation, 1-2-2 Akasaka, Minato-ku, Tokyo 107-0052, Japan.

The year 2025 marks the 50th anniversary of The Nippon Foundation and Sasakawa Health Foundation’s support for national leprosy programmes through the World Health Organization (WHO).

Over the past five decades, our foundations have provided cumulative support of approximately 200 million United States dollars (US$), including US$ 50 million for the free distribution of multidrug therapy from 1995 to 1999. We continue to assist around 40 countries annually, focusing on those with a high burden of leprosy, and our funding still constitutes the majority of WHO’s budget for leprosy.

Private foundations rarely sustain such long-term support for a single disease, but The Nippon Foundation and Sasakawa Health Foundation remain committed, despite the declining global priority given to leprosy in recent years.

Leprosy is a curable disease, and since the introduction of WHO-recommended multidrug therapy in the 1980s, the number of patients has drastically declined. In 2023, 182 815 new cases were diagnosed,^1^ in contrast to an estimated prevalence of 12 million patients worldwide in 1985.^2^

In 1991, the World Health Assembly adopted Resolution WHA 44.9 *Leprosy*, setting a target of reducing registered cases to below 1 per 10 000 people globally.^3^ This goal was achieved by the end of 2000, and by 2010, it had also been achieved at the national level by almost all countries except Brazil and some island states with fewer than 1 million inhabitants. However, leprosy has not disappeared.

After achieving elimination, many countries deprioritized leprosy, considerably reducing budgets and personnel, which resulted in hampered early case detection efforts and in difficulties assessing actual case numbers. In many countries, the number of new cases has remained static for over a decade.

Leprosy is one of the oldest known infectious diseases, and throughout its long history, those with leprosy have faced debilitating prejudice and violations of their human rights. In response, the Member States unanimously adopted in 2010 the United Nations Resolution A/RES/65/215 *Elimination of discrimination against persons affected by leprosy and their family members*,^4^ along with accompanying principles and guidelines.^5^ However, effective measures have yet to be fully implemented as these documents are not legally binding.

As of 2025, 139 discriminatory laws related to leprosy still exist in 24 countries (108 of these laws are in place in India).^6^ In addition, many traditional unwritten customs and practices remain a source of discrimination.^7^

WHO’s *Towards zero leprosy: global leprosy (Hansen’s disease) strategy 2021–2030*^8^ outlines key strategic pillars to achieve zero leprosy: implement integrated, country-owned zero leprosy roadmaps in all endemic countries; scale up leprosy prevention alongside integrated active case detection; manage leprosy and its complications and prevent new disability; and combat stigma and ensure human rights are respected.

The strategy aims to achieve several targets at global level, with each country setting its own targets: a 70% reduction in the annual number of new cases detected; a 90% reduction in the new cases per million population with grade-2 (visible) disability; 120 countries reporting zero new autochthonous cases (up from 45 countries in 2019); and the amendment or elimination of remaining discriminatory laws. 

In 2023, WHO introduced new numerical targets under the *Leprosy elimination monitoring tool*.^9^ These targets include achieving interruption of transmission (no new autochthonous cases among children for five consecutive years) and elimination of disease (no new autochthonous cases for three years and no child cases for five years). To reach these goals, WHO promotes preventive measures such as post-exposure prophylaxis and the integration of leprosy programmes with other neglected tropical diseases.

The Nippon Foundation and Sasakawa Health Foundation see leprosy as a symbol of humanity’s long-standing fight against infectious diseases; eliminating it would represent a remarkable achievement. Furthermore, the disease intersects with many global challenges highlighted in the United Nations sustainable development goals, so efforts against it also address broader challenges.

For example, many people affected by leprosy belong to impoverished communities. Within these, women and children often face harmful and wrongful stereotyping and structural violence.^10^ This situation makes leprosy not only a health issue, but also a matter of poverty, education, gender equality, equity, peace and justice.

Leaving no one behind requires tackling leprosy and the social vulnerability of those affected. The lessons learnt from efforts to end leprosy will undoubtedly help address broader global challenges. That’s why The Nippon Foundation and Sasakawa Health Foundation have been maintaining the partnership with WHO for 50 years and our commitment remains unwavering. Despite the relatively small number of cases compared to other diseases, leprosy must not be forgotten.
